# Bacterial contamination of sterile angiographic work environments during animal studies

**DOI:** 10.1371/journal.pone.0311112

**Published:** 2024-11-21

**Authors:** Christiane Franz, Lara Bender, Thorsten Sichtermann, Jan Minkenberg, Andrea Stockero, Christoph Dorn, Farzaneh Yousefi, Dimah Hasan, Manuela Schmiech, Rebecca May, Sophia Honecker, Sebastian Lemmen, Omid Nikoubashman, Martin Wiesmann, Hani Ridwan

**Affiliations:** 1 Department of Diagnostic and Interventional Neuroradiology, University Hospital RWTH Aachen, Aachen, Germany; 2 Division of Infection Control and Infectious Diseases, University Hospital RWTH Aachen, Aachen, Germany; 3 Institute of Infection Control and Infectious Diseases, Aachen, Germany; Osaka University Graduate School of Medicine, JAPAN

## Abstract

Bacterial contamination of angiographic materials and fluids has been shown to occur during human angiographic procedures. Angiographic examinations and experiments must be performed under sterile conditions to avoid complications due to contamination and possible subsequent infections. However, data regarding the frequency and the clinical consequences are limited. Our aim was to investigate the incidence of bacterial contamination during experimental angiographies. We tested angiographic fluids, syringes and endovascular materials from the angiographic supply tables for bacterial contamination, collecting 252 samples during 18 experimental angiographies in pigs. After sterile filtration, samples were cultured on media, and individual colony-forming units were identified by matrix-assisted laser desorption ionization–time of flight mass spectrometry. Contamination was detected in the majority of samples (60%). There was no angiography in which all samples remained sterile. The highest contamination rates (94%) were found in fluids from the working bowls and on the outer surface of syringes (85%) at the end of angiography. At this time, working bowls were significantly more frequently and extensively contaminated than the control bowls. Among the samples, the frequency and extent of contamination increased with the duration of the experimental angiographic procedures. Our findings show that bacterial contamination during angiography is common and the manipulation of endovascular working materials as well as the duration of angiographic procedures both increase bacterial contamination. While the clinical impact on the laboratory animal remains unclear, the quality of biomedical research mandates that efforts to minimize bacterial contamination should be taken as far as possible.

## Introduction

Pigs are widely accepted animal models because of their physiological similarities with humans, including body size, hematologic parameters and anatomical features [1–3]. They are particularly suitable for angiographic studies, such as in neuroradiological and cardiological research fields [1, 4, 5]. Angiographic examinations and experiments must be performed under sterile conditions to avoid complications due to contamination and possible subsequent infections. Despite the sterile preparation of the angiographic supply tables, a recent study revealed that bacterial contamination can take place in fluid-containing bowls used during angiography [6, 7]. The risk of bacterial contamination during human angiographies was investigated in some older studies, but systematic studies investigating this risk in animal experiments are lacking [8–10]. The bacterial contamination of angiographic materials during experimental angiographic animal studies may, in case of bacterial transmission and infection, compromise animal welfare and adversely affect the quality of biomedical research. Therefore, the objective of this study was to assess the actual bacterial contamination of the angiographic injection fluids, syringes and endovascular materials. To the best of our knowledge, there are no studies investigating this topic so far.

## Material and methods

### Animals and experimental procedures

All animals were part of another endovascular study with nine female Aachen minipigs (Gerd Heinrichs, Heinsberg-Karken, Deutschland; mean weight of 42.3kg ± 3.75kg (mean ± SD); age of 17–21 months) that underwent two angiographies resulting in a total of 18 angiographies. The animal experiments were conducted in accordance with the German Animal Welfare Law and the EU Directive 2010/63/EU after approval of the experimental protocol by the governmental animal care and use committee (Landesamt für Natur, Umwelt und Verbraucherschutz (LANUV) Nordrhein-Westfalen, Recklinghausen, Germany) the corresponding approval number being AZ-81-02.04.2019.A412. The conditions of animal housing complied with the requirements of Appendix III of EU Directive 2010/63/EU and the Appendix of the European Agreement of March 18, 1986. Institutional guidelines for animal welfare and experimental implementation were followed.

Animal handling, anesthesia and euthanasia were carried out as previously described [11]. The preparation of the pigs’ inguinal region included alternating scrubs with disinfectant soap (Baktolin, Paul Hartmann AG, Heidenheim, Germany). Subsequently, a final application of a disinfectant solution (Cutasept F, Paul Hartmann AG, Heidenheim, Germany) was conducted two times with exposure times of at least one minute each. The experimenters performed a presurgical hand scrub and disinfected their hands thoroughly with a disinfectant solution (Sterilium, Paul Hartmann AG, Heidenheim, Germany), while the manufacturers’ recommendations regarding exposure times were followed. Sterile surgical gloves, gowns, caps, and face masks were always used. The angiographic procedures were performed by a team of three highly experienced neuroradiologists. The neuroradiologists did not change within an angiography. The interventional procedures were part of a scientific endovascular study that did not serve training purposes, and were performed in the operation theater of the central laboratory animal facility of our institution.

The animals received a single shot of antibiotics (750 mg Cefuroxim, Hikma, London, England) during the first angiography. Afterwards, all pigs were kept at the institute for three or six months as part of the superordinate study. Due to the study design of the superordinate study, the second angiography was performed as final control at the end of the experiments. For this reason, no further antibiotics were administered as the animals were euthanized subsequently. During the chronic course of the study, the animals were regularly assessed using score sheets according to the superordinate study. This score sheet included criteria such as apathia, anorexia, or reduced food intake, which could be induced by bacteremia. However, these symptoms can also arise due to other differential diagnoses. The score sheet did not include specific criteria regarding bacteremia, (e.g., bloodcultures or C-reactive protein), because this was not part of the superordinate study and would have meant additional blood sampling for the animals.

### Experimental setup

In a standardized approach, we examined neuroangiographic fluids (0.9% sterile sodium chloride solution, Fresenius, Bad Homburg vor der Höhe, Germany) and angiographic material (i.e., catheters, guide wires, syringes) derived from a total of 18 angiographies of an animal study. A standard diagnostic supply table was prepared at the beginning of the experiments including a working bowl and a control bowl. Both bowls were filled with sterile saline and the working bowl was used to store endovascular materials during the angiographic experiments, while the control bowl remained in the sterile field during angiographic experiments without manipulation or placement of any materials. Metal cups were used to provide saline and contrast medium for injection during angiography. Samples were taken at the beginning of angiography, directly after preparation of the angiographic supply table. The samples at the end of the angiography were taken immediately after completion of the endovascular procedure. We collected the following samples during each angiography: Fluid from the control bowl at the beginning (control bowl_0_) and at the end of angiography (control bowl_1_), as well as fluid from the working bowl at the beginning (working bowl_0_) and at the end of angiography (working bowl_1_), 50 ml each. At the end of each angiography, the three angiographic 10 ml syringes (B. Braun, Melsungen, Germany) routinely used for angiographies in our department were filled with saline or contrast agent from the metal cup. These samples were taken to detect potential contamination of the fluids and were used directly to assess contamination of the inner surfaces of syringes. The syringes themselves, as well as tips and ends of the catheters and guidewires were also collected as samples. This resulted in a total of 14 samples for each angiography and a total of 252 samples of all angiographies. All samples were taken using sterile techniques and placed into sterile sample containers.

### Microbiological laboratory

Further sample processing was conducted under a laminar flow hood (HeraSafe KS, Thermo Scientific, Waltham, United States) according to a routine hospital hygiene protocol for the detection of bacterial contamination in fluids. The samples, which originally consisted of fluid (i.e., fluids from control bowls, working bowls, and content of syringes), were directly used for sterile filtration. The other samples (i.e., catheters, guide wires, and syringes) were each added with 500 ml sterile saline and left on a shaker for 20 min at 200 rpm (Unimax1010, Heidolph, Schwabach, Germany). For sterile filtration, an aspiration system was loaded with sterile filters and funnels (Cellulose Nitrate 0.2μm and Biosart 250 Funnels Sartorius, Göttingen, Germany). The filters were then individually transferred to a microbiological culture medium (TSAB Agar (Tryptone soya agar with sheep blood), Oxoid, Wesel, Germany) and remained there for the entire incubation period. Incubation of the culture media was performed for 48 hours in an incubator (T = 37°C, CO^2^ = 0.8%, Heracell, Heraeus, Hanau, Germany). Subsequently, colonies were counted and assessed macroscopically. Microscopic assessment was conducted by classical Gram staining. When necessary, single strains were incubated for an additional 24 hours. Species identification was performed using matrix-assisted laser desorption ionization–time of flight mass spectrometry (MALDI-ToF MS) (Daltonik MALDI Biotyper, Bruker, Billerica, United States). During the interpretation of the MALDI-ToF MS results, a log (score) of ≥ 2.0 was applied as the threshold for a match at the species level.

### Statistical analysis

Continuous variables are presented as median and interquartile range (IQR), after Shapiro-Wilk test showed that data were not normally distributed. When comparing the contamination rates, we performed Chi-square tests. Paired Wilcoxon or Mann-Whitney U-tests were used for comparisons with respect to the number of colony forming units. All tests were two-sided. P-values under the α-level of 0.05 were defined as significant. The Spearman Rho correlation coefficient was calculated to investigate the relationship between variables. All statistical analyses were performed with SPSS 28 software (IBM, San Jose, California, USA).

## Results

According to our score sheet assessment, none of the animals showed general clinical signs of inflammation (local or systemic) that could have been associated with angiography-induced bacteremia.

A total of 252 samples were taken. The duration of angiographies and the number of colony forming units were not normally distributed (p < 0.005). The angiographies (n = 18) had a mean duration of 3.67 h (median, 3.63 h; IQR, 1.92–5.75 h). Due to the high complexity of the endovascular study, the angiographies required relatively long procedure times.

We evaluated the frequency of contaminated samples (contamination rate) and the number of detected colony-forming units (CFU) (extent of contamination). All bacteriological results can be found in the supporting information file (S1 Table bacteriological results).

### Quantitative analysis

There was no angiography in which all samples remained sterile. Of the 252 samples evaluated, a total of 150 were contaminated (60%) with a median of 1 CFU (IQR, 1–17) per contaminated sample. At the beginning of angiography, CFUs were detected in 11/18 (61%) of the working bowls with a median of 1 CFU (IQR, 0–2) and in 8/18 (44%) of the control bowls with a median of 0 CFUs (IQR, 0–11). There were no significant differences in the contamination rates between the control bowls and working bowls at the beginning of angiography. In the control bowls, the contamination rate was slightly lower in the beginning (c_0_ = 44%) compared to the end (c_1_ = 56%), but there was no statistically significant difference (p = 0.505). In working bowls, both the contamination rates (w_0_ = 61%, w_1_ = 94%) and the number of CFUs significantly increased when comparing the beginning with the end of angiography (p = 0.016 and p < 0.001, respectively). At the end of angiography, CFUs were detected in 17/18 (94%) of the working bowls with a median of 17 CFUs (IQR, 6–80). CFUs were detected in 10/18 (56%) of the control bowls with a median of 2 CFUs (IQR, 0–13). At the end of angiography, both the contamination rate and the number of CFUs were significantly higher in the working bowls than in the control bowls (p = 0.007 and p = 0.005, respectively).

At the end of angiography, CFUs were detected in fluid samples from the inner surfaces of syringes in 25/54 (46%) of the syringes with a median of 1 CFU (IQR, 1–9) and in 46/54 (85%) on the outer surface of syringes with a median of 30 CFUs (IQR, 7–113). Both the contamination rates and the number of CFUs were significantly higher on the outer surface of syringes than on the inner surface of syringes (p-values < 0.001). At the end of angiography, CFUs were detected in 8/18 (44%) samples from catheter tips with a median of 0 CFUs (IQR, 0–1) and in 11/18 (61%) samples from catheter ends with a median of 1 CFUs (IQR, 0–7).

**Fig 1:**
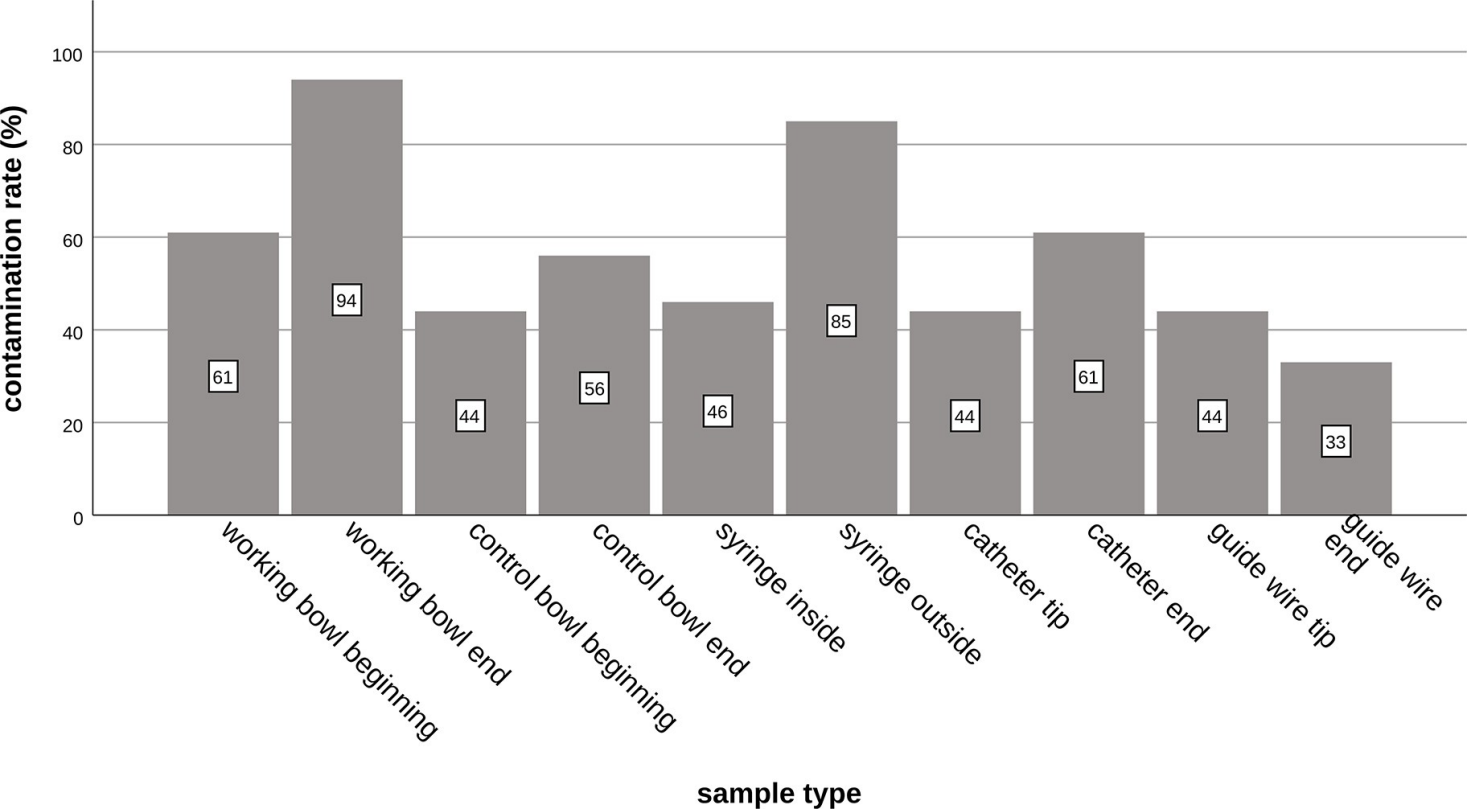
Contamination frequencies of the different sample types. The contamination rates of the different sample types show that the working bowls at the end of the angiography (94%) and the outside of the syringes (85%) are most frequently contaminated. Asterisks illustrate levels of significant differences: *(p < 0.05), ** (p < 0.01), and *** (p < 0.001).

**Fig 2:**
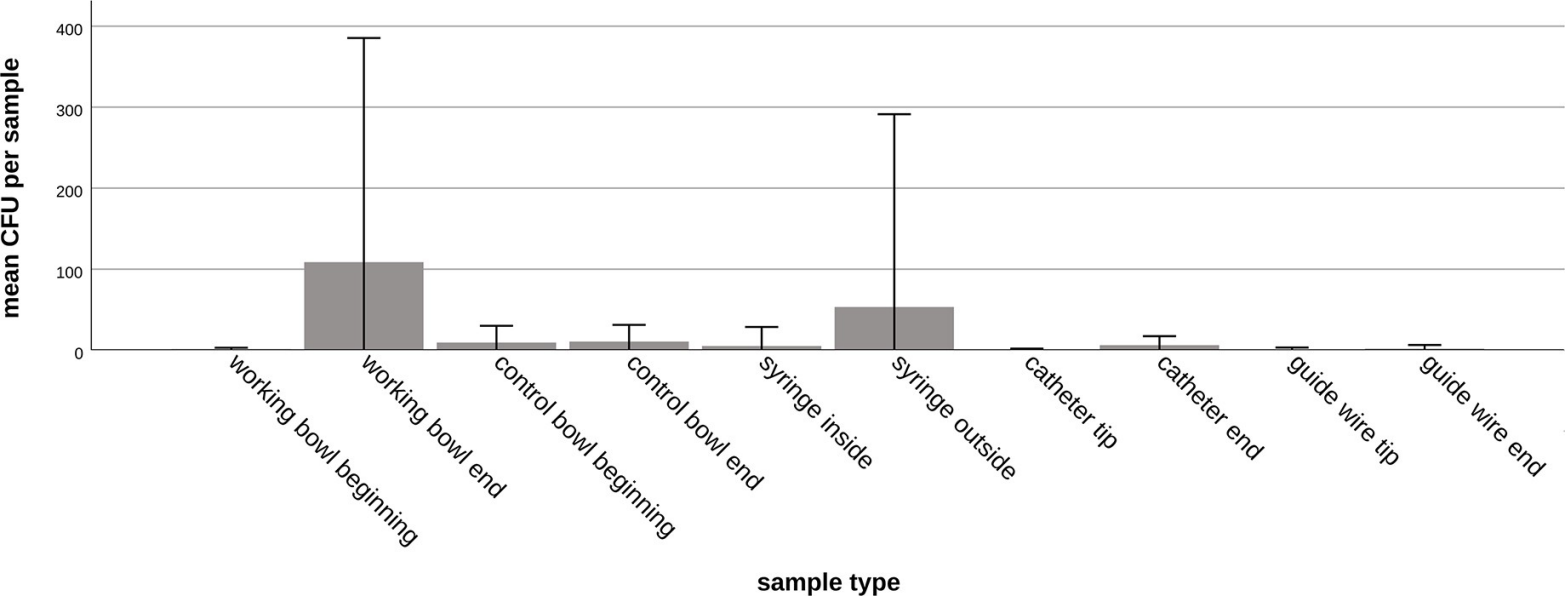
Numbers of colony-forming units in the different sample types. With regard to the number of CFUs found, the highest mean values per sample were found in the working bowls (mean, 108.7 CFUs) and on the outside of the syringes (mean, 159.6 CFUs) at the end of angiography. Error bars represent the standard deviation.

Samples from guide wire tips yielded positive cultures in 8/18 (44%) samples with a median of 0 CFUs (IQR, 0–1) and guide wire ends in 6/18 (33%) samples with a median of 0 CFUs (IQR, 0–1). In the samples from tips of endovascular materials (catheter tips and guide wire tips) contamination was detected in 16/36 (44%) samples whereas the ends of endovascular material (catheter ends and guide wire ends) yielded positive cultures in 17/36 (47%) of the samples. There were no statistically significant differences between the samples from the different endovascular material groups (catheter tips vs. guide wire tips, catheter ends vs. guide wire ends, catheter tips vs. guide wire ends, catheter ends vs. guide wire tips, tips vs. ends) (p-values > 0.095). The contamination rates of different sample types are illustrated in Fig 1, while numbers of CFUs are depicted in Fig 2.

#### Qualitative analysis of the bacterial spectrum

Qualitative analysis yielded a bacterial spectrum of 55 different species (Table 1). The bacterial spectrum consisted of environmental apathogenic bacteria and skin microbiota in the vast majority of cases: 92.2% were classified as environmental or skin germs, whereas 6.5% were classified as other gram-negative germs, and 1.3% were classified as potentially pathogenic germs.

**Table 1:** Bacterial spectrum found in the samples.

Germ species	Gram	Group
Acinetobacter spp.	negative	environmental or skin germs
Aerococcus viridans	positive	environmental or skin germs
Bacillus cereus	positive	environmental or skin germs
Bacillus pumilus	positive	environmental or skin germs
Bacillus spp.	positive	environmental or skin germs
Bacillus thermoaylovorans	positive	environmental or skin germs
Brachybacterium muris	positive	environmental or skin germs
Brevibacterium luteolum	positive	environmental or skin germs
Corynebacterium afermentans	positive	environmental or skin germs
Corynebacterium camporealensis	positive	environmental or skin germs
Corynebacterium confusum	positive	environmental or skin germs
Corynebacterium glutamicum	positive	environmental or skin germs
Corynebacterium mucifaciens	positive	environmental or skin germs
Corynebacterium spp.	positive	environmental or skin germs
Deinococcus wulumuqiensis	positive	environmental or skin germs
Dermacoccus nishinomyaensis	positive	environmental or skin germs
Dietzia natroemnaea	positive	environmental or skin germs
Kocuria palustris	positive	environmental or skin germs
Kocuria rhizophila	positive	environmental or skin germs
Kocuria spp.	positive	environmental or skin germs
Kytococcus spp.	positive	environmental or skin germs
Lysinibacillus spp.	positive	environmental or skin germs
Micrococcus luteus	positive	environmental or skin germs
Moraxella osloensis	negative	other gram-negative germs
Moraxella spp.	negative	other gram-negative germs
Paenibacillus urinalis	positive	environmental or skin germs
Pantoea agglomerans	negative	other gram-negative germs
Paracoccus yeei	negative	other gram-negative germs
Pseudomonas oryzihabitans	negative	other gram-negative germs
Pseudomonas stutzeri	negative	other gram-negative germs
Roseomonas mucosa	negative	other gram-negative germs
Rothia aerolata	positive	environmental or skin germs
Rothia koreensis	positive	environmental or skin germs
Rothia nasimurium	positive	environmental or skin germs
Staphylococcus arlettae	positive	environmental or skin germs
Staphylococcus aureus	positive	potentially pathogenic
Staphylococcus capitis	positive	environmental or skin germs
Staphylococcus chromogenes	positive	environmental or skin germs
Staphylococcus cohnii	positive	environmental or skin germs
Staphylococcus epidermidis	positive	environmental or skin germs
Staphylococcus haemolyticus	positive	environmental or skin germs
Staphylococcus hominis	positive	environmental or skin germs
Staphylococcus hyicus	positive	potentially pathogenic
Staphylococcus intermedius	positive	environmental or skin germs
Staphylococcus kloosii	positive	environmental or skin germs
Staphylococcus lentus	positive	environmental or skin germs
Staphylococcus lugdunensis	positive	potentially pathogenic
Staphylococcus pasteuri	positive	environmental or skin germs
Staphylococcus pettenkoferi	positive	environmental or skin germs
Staphylococcus saprophyticus	positive	environmental or skin germs
Staphylococcus sciuri	positive	environmental or skin germs
Staphylococcus simulans	positive	environmental or skin germs
Staphylococcus spp.	positive	environmental or skin germs
Staphylococcus warneri	positive	environmental or skin germs
Staphylococcus xylosus	positive	environmental or skin germs

*Staphylococcus spp*. (56.5%) and *Micrococcus spp*. (16.5%) were found most frequently. The following species were classified as potentially pathogenic: *Staphylococcus aureus*, *Staphylococcus lugdunensis*, *and Staphylococcus hyicus*.

In total, we detected 5659 CFUs in our study. Of these, only 18 CFUs were from potentially pathogenic species. Of those 18 CFUs, 2 CFUs of *Staphylococcus hyicus*, 3 CFUs of *Staphylococcus lugdunensis* and 13 CFUs of *Staphylococcus aureus* were detected.

#### Duration of angiography as an influencing factor

The longer the duration of the angiographic procedures, the more frequently samples were contaminated (p = 0.006). The uncontaminated samples were from angiographies with a mean duration of 3.33 h ± 1.88 h (median, 2.5 h; IQR, 1.92–5.75 h), while the contaminated samples were from angiographies with a mean duration of 3.90 ± 1.81 h (median, 5.00 h; IQR 2.00–5.75 h). The duration of the angiographic procedures had also a significant influence on the number of CFUs found. The longer the duration of examination, the more CFUs were detected. The Spearman-Rho correlation found (r = 0.633) was statistically significant (p = 0.005).

## Discussion

The main finding of our study was that at the end of experimental angiographies, bacterial contamination of angiographic materials and fluids was detected in 100% of the angiographies, although sterile precautions were followed.

Previous authors report that bacteremia occurs in 4–8% of angiographies, but is asymptomatic in most cases [8–10]. For example, after dental extraction bacteremia has been found in 100% of cases [12]. Although bacteremia is usually clinically inconsequential in immunocompetent patients, bacterial contamination has to be avoided during sterile procedures in human and veterinary medicine. Given the increasing complexity of neuroangiographic procedures, this imperative extends to laboratory animal experiments with regard to animal welfare and the reliability of biomedical research results. Although the presence of bacterial contamination does not invariably lead to subsequent bacteremia and clinical illness, the potential impact of contamination during angiographies cannot be underestimated in angiographic animal studies and possibly represents a confounding factor. Systemic animal studies on this topic are lacking, to the best of our knowledge, but insights from human medicine indicate that sterile fluids used in angiography can be contaminated with both bacteria and particles [6, 13].

On the basis of these observations, we hypothesized that during angiographies, bacteria are most likely transmitted through injections of contaminated fluids, the insertion of contaminated endovascular materials (e.g., catheters or guide wires), or the use of contaminated syringes. Importantly, our results show that at the end of angiography, the contamination rate (94%) and the number of CFUs in the working bowls were significantly higher than those in the control bowls (contamination rate 56%). Moreover, we found significantly higher contamination rates and numbers of CFUs in the working bowls at the end of angiography than at the beginning (p < 0.001), indicating that the manipulation of the working bowls during angiographic procedures significantly contribute to the contamination of fluid-containing working bowls. Relevant literature provides possible causes of contamination such as surface contamination within the operation theater, contact with contaminated fluids or glove perforations, or airborne transmission [14–17]. Furthermore, we found a positive and significant correlation between the duration of angiography and the total number of CFUs (Spearman correlation coefficient = 0.633, p < 0.005). Possible explanations may include a higher number of manipulations and contact to gloves of the operator during longer procedure times. However, this assumption would have to be proven in future studies.

During angiographies, syringes are used to inject saline and contrast agents. To assess their role in bacterial transmission, we analyzed the inner and outer surfaces of the syringes. Based on findings from previous studies, we expected a certain degree of contamination on the outer surface of the syringes, possibly originating from perforated gloves or fluid-containing bowls [6, 14, 15]. Moreover, a previous study of Wiesmann et al. also dealt with contamination of syringes in neuroangiographic settings with human patients and reported contamination of inner surfaces of syringes in 23% and contamination of outer surfaces in 91% of the cases [7]. Even though our recent study included a broader spectrum of angiographic materials, e.g., catheters and fluid-containing bowls, we were able to confirm the earlier findings of Wiesmann et al. regarding the contamination of syringes in experimental angiographies with pigs. In both studies the contamination of the outer surfaces of the syringes was significantly higher than the contamination of the inner surface of syringes [7]. The outer surface contamination of syringes does not necessarily need to result in transmission of bacteria into the animal’s bloodstream. In contrast, bacterial transmission is likely, and subsequent bacteremia is expected to be higher if the inner surface of the syringes is contaminated. In fact, nearly all syringes were contaminated on the outside (85%). Surprisingly, a considerable proportion of the inner surfaces within the syringes was also contaminated (46%). We hypothesize that this contamination of fluids may be a result from the use of separate open metal cups for holding injection fluids. On the basis of the results presented here, we therefore recommend the use of closed-line systems for saline and contrast agents during angiographic procedures, as proposed by Nikoubashman et al. [13]. Although closed-line systems are available for angiographic applications in cardiology, radiology, or neuroradiology in human medicine, angiographic practices vary considerably between different countries and hospitals, and open bowls are often preferred to provide contrast agents during angiographies [6]. Consequently, our recommendation applies to sterile procedures in laboratory animals, where the same measures should be taken as in angiographic settings with human patients.

Given the workflow during angiographic procedures, one could expect that the ends of endovascular materials would be more frequently contaminated than the tips of those materials. We found that catheter ends were most frequently contaminated (61%), although no significant differences were observed compared with other endovascular material tips or ends (p-values ≥ 0.095). These results indicate that the contamination of the endovascular materials may rather be due to their storage in the working bowls, which were nearly almost contaminated, than to handling during the procedure.

Surprisingly and despite of sterile preparation, we detected a certain degree of contamination at the beginning of the angiographic procedures in our study. We believe that these contaminations were caused by small air turbulences during the filling of the fluid-containing bowls. However, the contamination rates and CFU counts of the control bowls at the beginning did not significantly increase compared to those at the end of the angiographies (p = 0.505). Although airborne transmission does not seem to contribute to the increase of contamination rate and frequency during the course of angiographic procedures, it seems to play a role in the beginning of angiography.

The spectrum of bacterial species found was predominantly caused by apathogenic environmental contamination. Out of a total of 5659 CFUs, only 18 potentially pathogenic CFUs were found, this proportion appears to be extremely low.

In summary, a considerable bacterial burden seems to occur in sterile experimental angiographies, which is almost entirely caused by environmental or non-pathogenic bacteria. Moreover, there is also a low contamination by originated potentially pathogenic bacteria. In angiographic animal studies, the contamination rate and the risk of inducing bacteremia could be underestimated and possibly have negative consequences. On the one hand, contamination during experimental angiographies could interfere with biomedical research results. On the other hand, unrecognized infections could reduce animal welfare and increase animal losses. Furthermore, immunosuppressed large animal models, currently used in various biomedical research areas [18], are considerably more sensitive to infections and may have a higher risk of developing clinical manifestations as a result of bacteremia.

## Limitations

We are well aware of the limitations of this study, as we did not investigate different transmission routes of bacterial contamination to the animals’ bloodstream. Furthermore, we did not aim to investigate bacteremia, because this would have required additional blood sampling from the animals. Finally, the clinical impact of bacteremia caused by angiographic procedures was not investigated and could be a target for future studies.

## Conclusions

In conclusion, our findings show that bacterial contamination during experimental angiographies involving laboratory pigs is common and the manipulation of endovascular working materials as well as the duration of angiographic procedures both increase bacterial contamination. While the clinical impact on the laboratory animal remains unclear, the quality of biomedical research mandates that efforts to minimize bacterial contamination should be taken as far as possible.

## Supporting information

S1 Table Bacteriological results. (PDF)
