# Supplementary material for: Bacterial contamination of sterile angiographic work environments during animal studies
Source: PLoS One. 2024 Nov 21;19(11):e0311112. doi: 10.1371/journal.pone.0311112 (PMC11581324; doi:10.1371/journal.pone.0311112)
Supplement: S1 Table — (PDF) [file pone.0311112.s001.pdf]

Angiography no.	Sample	No. Of CFUs	Species
1	working bowl beginning	1	Staphylococcus pettenkoferi
1	working bowl beginning	1	Staphylococcus cohnii
1	control bowl beginning	4	Staphylococcus haemolyticus
1	control bowl beginning	1	gram-negative cocci
1	control bowl beginning	1	Staphylococcus cohnii
1	control bowl beginning	3	Rothia nasimurium
1	control bowl beginning	6	Staphylococcus pasteuri
1	control bowl beginning	5	gram-negative cocci
1	control bowl beginning	1	gram-positive cocci
1	working bowl end	2	Deinococcus wulumuqiensis
1	working bowl end	1	Corynebacterium glutamicum
1	working bowl end	6	Staphylococcus haemolyticus
1	working bowl end	2	Corynebacterium confusum
1	working bowl end	35	Staphylococcus capitis
1	working bowl end	30	Staphylococcus epidermidis
1	control bowl end	1	Bacillus cereus
1	control bowl end	7	Staphylococcus cohnii
1	control bowl end	1	Rothia nasimurium
1	control bowl end	1	Staphylococcus chromogenes
1	control bowl end	3	Staphylococcus pettenkoferi
1	control bowl end	1	Staphylococcus simulans
1	control bowl end	4	Staphylococcus epidermidis
1	syringe inside	5	Staphylococcus epidermidis
1	syringe outside	1	Micrococcus luteus
1	syringe outside	3	Staphylococcus pettenkoferi
1	syringe outside	1	Bacillus thermoaylovorans
1	syringe outside	1	Staphylococcus warneri
1	syringe outside	1	Paracoccus yeei
1	syringe outside	1	Staphylococcus pettenkoferi
1	catheter end	1	Staphylococcus epidermidis
2	control bowl beginning	1	Micrococcus luteus
2	working bowl end	13	Staphylococcus epidermidis
2	working bowl end	3	Staphylococcus pettenkoferi
2	working bowl end	1	Staphylococcus cohnii
2	working bowl end	1	Staphylococcus hyicus
2	syringe inside	1	Staphylococcus haemolyticus
2	syringe outside	20	Staphylococcus haemolyticus
2	syringe outside	5	Staphylococcus pettenkoferi
2	syringe outside	1	Staphylococcus capitis
2	syringe outside	2	Staphylococcus cohnii
2	syringe outside	8	Staphylococcus pettenkoferi
2	syringe outside	5	Micrococcus luteus
2	syringe outside	21	Staphylococcus pettenkoferi
2	syringe outside	2	Staphylococcus haemolyticus
2	syringe outside	2	Staphylococcus cohnii
2	syringe outside	1	Staphylococcus hyicus
2	syringe outside	3	Corynebacterium afermentans
2	catheter tip	1	Staphylococcus haemolyticus
2	catheter tip	1	Staphylococcus pettenkoferi

2	guide wire tip	1	Staphylococcus warneri
2	guide wire tip	2	Staphylococcus pettenkoferi
2	catheter end	1	Micrococcus luteus
2	catheter end	4	Staphylococcus pettenkoferi
2	catheter end	4	Staphylococcus capitis
2	catheter end	1	Bacillus spp.
2	catheter end	1	Staphylococcus cohnii
3	control bowl beginning	9	Staphylococcus aureus
3	control bowl beginning	2	Staphylococcus epidermidis
3	working bowl end	100	Kocuria spp.
3	working bowl end	1	Corynebacterium camporealensis
3	working bowl end	1	Moraxella spp.
3	working bowl end	2	Kytococcus spp.
3	working bowl end	9	Staphylococcus lentus
3	working bowl end	111	Staphylococcus cohnii
3	control bowl end	4	Staphylococcus aureus
3	control bowl end	1	Micrococcus luteus
3	control bowl end	1	Staphylococcus capitis
3	control bowl end	2	Staphylococcus epidermidis
3	syringe inside	3	Staphylococcus cohnii
3	syringe inside	5	Kocuria spp.
3	syringe inside	1	gram-positive cocci
3	syringe inside	20	gram-positive cocci
3	syringe inside	17	Staphylococcus sciuri
3	syringe inside	32	Staphylococcus cohnii
3	syringe inside	102	Kocuria spp.
3	syringe inside	2	Staphylococcus cohnii
3	syringe outside	38	Staphylococcus cohnii
3	syringe outside	10	Staphylococcus haemolyticus
3	syringe outside	37	Kocuria spp.
3	syringe outside	1	Staphylococcus kloosii
3	syringe outside	1	Staphylococcus capitis
3	syringe outside	12	Staphylococcus cohnii
3	syringe outside	7	Staphylococcus pettenkoferi
3	syringe outside	1	Staphylococcus haemolyticus
3	syringe outside	3	Staphylococcus spp.
3	syringe outside	28	Kocuria spp.
3	syringe outside	2	Staphylococcus capitis
3	syringe outside	34	Staphylococcus cohnii
3	syringe outside	6	Staphylococcus haemolyticus
3	syringe outside	4	Rothia koreensis
3	syringe outside	2	Staphylococcus spp.
3	syringe outside	72	Kocuria spp.
3	syringe outside	1	Aerococcus viridans
3	catheter end	1	Staphylococcus cohnii
4	working bowl end	3	Staphylococcus intermedius
4	working bowl end	2	Corynebacterium confusum
4	working bowl end	44	Staphylococcus pettenkoferi
4	working bowl end	4	Micrococcus luteus
4	working bowl end	70	Staphylococcus haemolyticus

4	working bowl end	65	Staphylococcus cohnii
4	control bowl end	5	Staphylococcus capitis
4	syringe inside	1	Staphylococcus hominis
4	syringe inside	1	Staphylococcus pettenkoferi
4	syringe outside	1	Staphylococcus cohnii
4	syringe outside	1	Staphylococcus capitis
4	syringe outside	1	Staphylococcus capitis
4	syringe outside	127	Staphylococcus pettenkoferi
4	syringe outside	33	Staphylococcus cohnii
4	syringe outside	1	Pseudomonas oryzihabitans
4	syringe outside	1	gram-positive rods
4	catheter tip	3	Moraxella osloensis
4	catheter tip	1	gram-positive cocci
4	guide wire end	13	Staphylococcus cohnii
4	guide wire end	2	Micrococcus luteus
4	guide wire end	2	Staphylococcus pettenkoferi
5	control bowl beginning	2	Corynebacterium camporealensis
5	working bowl end	256	Staphylococcus cohnii
5	working bowl end	389	Staphylococcus haemolyticus
5	working bowl end	542	Staphylococcus chromogenes
5	control bowl end	7	Staphylococcus epidermidis
5	control bowl end	1	Kocuria rhizophila
5	control bowl end	1	Corynebacterium confusum
5	control bowl end	1	Staphylococcus spp.
5	control bowl end	2	gram-negative cocci
5	syringe inside	9	Staphylococcus cohnii
5	syringe inside	22	Kocuria spp.
5	syringe inside	1	Staphylococcus epidermidis
5	syringe inside	1	Micrococcus luteus
5	syringe inside	1	Staphylococcus cohnii
5	syringe inside	2	Kocuria spp.
5	syringe outside	2	Kocuria spp.
5	syringe outside	3	Staphylococcus hominis
5	syringe outside	6	Staphylococcus capitis
5	syringe outside	5	Staphylococcus cohnii
5	syringe outside	1	Brachybacterium muris
5	syringe outside	100	Corynebacterium mucifaciens
5	syringe outside	6	Staphylococcus hominis
5	syringe outside	1	Micrococcus luteus
5	syringe outside	8	Staphylococcus cohnii
5	syringe outside	7	Kocuria spp.
5	syringe outside	50	Corynebacterium mucifaciens
5	catheter end	14	Staphylococcus cohnii
5	catheter end	14	Kocuria spp.
5	catheter end	1	gram-positive cocci
5	guide wire end	4	Staphylococcus cohnii
5	guide wire end	7	Kocuria spp.
6	working bowl beginning	1	Corynebacterium camporealensis
6	working bowl beginning	1	Moraxella osloensis
6	control bowl beginning	12	Staphylococcus haemolyticus

6	control bowl beginning	4	Staphylococcus chromogenes
6	control bowl beginning	5	Staphylococcus cohnii
6	control bowl beginning	13	Staphylococcus pettenkoferi
6	control bowl beginning	4	Corynebacterium camporealensis
6	working bowl end	3	Micrococcus luteus
6	working bowl end	1	Staphylococcus haemolyticus
6	working bowl end	2	Staphylococcus chromogenes
6	working bowl end	3	Staphylococcus saprophyticus
6	working bowl end	1	Staphylococcus cohnii
6	working bowl end	4	gram-positive cocci
6	working bowl end	2	Moraxella spp.
6	control bowl end	3	gram-positive cocci
6	control bowl end	3	Staphylococcus chromogenes
6	control bowl end	1	Rothia nasimurium
6	control bowl end	1	Staphylococcus cohnii
6	syringe inside	1	Staphylococcus hominis
6	syringe outside	1	gram-positive cocci
6	syringe outside	1	Brevibacterium luteolum
6	syringe outside	1	Micrococcus luteus
6	syringe outside	1	Staphylococcus cohnii
6	syringe outside	1	Moraxella spp.
6	catheter tip	1	Acinetobacter lwoffii
6	guide wire tip	1	Staphylococcus epidermidis
6	catheter end	3	Staphylococcus hominis
6	catheter end	1	gram-positive cocci
6	guide wire end	1	Micrococcus luteus
7	working bowl beginning	1	Staphylococcus hominis
7	control bowl beginning	4	Staphylococcus haemolyticus
7	control bowl beginning	4	Staphylococcus cohnii
7	control bowl beginning	1	Kocuria palustris
7	control bowl beginning	2	Corynebacterium camporealensis
7	working bowl end	4	Staphylococcus haemolyticus
7	working bowl end	3	Corynebacterium camporealensis
7	working bowl end	3	Kocuria rhizophila
7	working bowl end	13	Pseudomonas stutzeri
7	working bowl end	11	Staphylococcus hominis
7	working bowl end	6	Staphylococcus chromogenes
7	working bowl end	1	Rothia nasimurium
7	working bowl end	10	gram-negative cocci
7	syringe inside	1	Staphylococcus epidermidis
7	syringe outside	6	Staphylococcus epidermidis
7	syringe outside	1	Acinetobacter lwoffii
7	syringe outside	1	Staphylococcus haemolyticus
7	syringe outside	2	Kocuria palustris
7	syringe outside	1	Staphylococcus epidermidis
7	syringe outside	1	Micrococcus luteus
7	guide wire tip	1	Staphylococcus epidermidis
7	catheter end	1	Staphylococcus saprophyticus
8	working bowl beginning	3	Staphylococcus cohnii
8	working bowl end	7	Staphylococcus haemolyticus

8	working bowl end	11	Corynebacterium camporealensis
8	working bowl end	17	Staphylococcus warneri
8	working bowl end	2	Moraxella spp.
8	control bowl end	17	Corynebacterium confusum
8	control bowl end	17	Staphylococcus pettenkoferi
8	control bowl end	2	Staphylococcus chromogenes
8	control bowl end	2	Rothia aerolata
8	syringe inside	1	Dermacoccus nishinomyaensis
8	syringe inside	1	Staphylococcus cohnii
8	syringe outside	1	Corynebacterium confusum
8	syringe outside	1	Staphylococcus haemolyticus
8	syringe outside	1	Pseudomonas stutzeri
8	catheter tip	1	Kocuria rhizophila
8	catheter end	1	Micrococcus luteus
8	catheter end	1	Staphylococcus sciuri
9	working bowl beginning	1	gram-positive cocci
9	control bowl beginning	2	Staphylococcus hominis
9	working bowl end	4	Moraxella osloensis
9	working bowl end	7	Staphylococcus haemolyticus
9	working bowl end	2	Staphylococcus capitis
9	working bowl end	7	Staphylococcus epidermidis
9	working bowl end	7	Staphylococcus cohnii
9	control bowl end	2	Moraxella osloensis
9	control bowl end	3	Kocuria spp.
9	control bowl end	2	Staphylococcus haemolyticus
9	control bowl end	9	Staphylococcus saprophyticus
9	syringe inside	1	Corynebacterium camporealensis
9	syringe inside	3	gram-positive cocci
9	syringe inside	1	gram-positive cocci
9	syringe inside	2	Staphylococcus capitis
9	syringe inside	1	Staphylococcus cohnii
9	syringe inside	2	Pantoea agglomerans
9	syringe outside	422	Staphylococcus haemolyticus
9	syringe outside	516	Corynebacterium confusum
9	syringe outside	463	Staphylococcus cohnii
9	syringe outside	351	Staphylococcus lentus
9	syringe outside	41	Corynebacterium camporealensis
9	syringe outside	11	gram-positive rods
9	syringe outside	6	Staphylococcus cohnii
9	syringe outside	1	Staphylococcus haemolyticus
9	syringe outside	16	gram-positive cocci
9	syringe outside	2	gram-positive cocci
9	syringe outside	3	Staphylococcus haemolyticus
9	syringe outside	1	Corynebacterium camporealensis
9	syringe outside	1	Moraxella osloensis
9	syringe outside	2	gram-positive cocci
9	syringe outside	3	Staphylococcus cohnii
9	syringe outside	3	Staphylococcus lugdunensis
9	syringe outside	1	Corynebacterium confusum
9	syringe outside	1	gram-positive cocci

9	syringe outside	1	Paenibacillus urinalis
9	guide wire tip	2	Staphylococcus capitis
9	catheter end	3	Staphylococcus haemolyticus
9	catheter end	2	Kocuria spp.
10	working bowl beginning	1	Staphylococcus capitis
10	control bowl beginning	33	Staphylococcus pettenkoferi
10	control bowl beginning	39	Staphylococcus haemolyticus
10	control bowl beginning	1	gram-positive cocci
10	control bowl beginning	2	Staphylococcus simulans
10	control bowl beginning	7	Staphylococcus pettenkoferi
10	working bowl end	1	Dietzia natroemnaea
10	working bowl end	1	Staphylococcus haemolyticus
10	working bowl end	4	Staphylococcus pettenkoferi
10	control bowl end	46	Staphylococcus pettenkoferi
10	control bowl end	30	Staphylococcus pettenkoferi
10	control bowl end	5	Staphylococcus pettenkoferi
10	control bowl end	2	Staphylococcus arlettae
10	syringe inside	1	Staphylococcus epidermidis
10	syringe outside	4	Staphylococcus xylosus
10	syringe outside	1	Moraxella spp.
10	syringe outside	1	Staphylococcus xylosus
10	syringe outside	14	Rothia nasimurium
10	syringe outside	5	Staphylococcus pettenkoferi
10	syringe outside	2	Rothia nasimurium
10	syringe outside	1	Staphylococcus xylosus
10	syringe outside	2	Moraxella osloensis
10	syringe outside	3	Staphylococcus capitis
10	syringe outside	1	Staphylococcus lentus
10	syringe outside	5	Staphylococcus hominis
10	syringe outside	1	Staphylococcus pettenkoferi
10	catheter tip	2	Staphylococcus epidermidis
10	catheter end	4	gram-positive cocci
10	catheter end	3	Staphylococcus xylosus
10	catheter end	19	Rothia nasimurium
10	catheter end	7	Rothia nasimurium
10	catheter end	1	gram-positive cocci
10	catheter end	3	Staphylococcus xylosus
10	catheter end	1	Rothia nasimurium
10	guide wire end	1	Micrococcus luteus
11	working bowl beginning	1	Staphylococcus pettenkoferi
11	working bowl end	28	Staphylococcus pettenkoferi
11	working bowl end	14	Staphylococcus haemolyticus
11	working bowl end	26	Corynebacterium camporealensis
11	working bowl end	7	Staphylococcus chromogenes
11	working bowl end	8	Staphylococcus arlettae
11	working bowl end	4	Staphylococcus pettenkoferi
11	working bowl end	3	Staphylococcus xylosus
11	syringe inside	3	Staphylococcus pettenkoferi
11	syringe inside	3	Staphylococcus pettenkoferi
11	syringe inside	1	Staphylococcus pettenkoferi

11	syringe inside	1	gram-positive cocci
11	syringe outside	3	Staphylococcus haemolyticus
11	syringe outside	8	Staphylococcus pettenkoferi
11	syringe outside	14	Staphylococcus capitis
11	syringe outside	21	Staphylococcus pettenkoferi
11	syringe outside	2	Staphylococcus xylosus
11	syringe outside	5	Staphylococcus chromogenes
11	syringe outside	8	Corynebacterium confusum
11	syringe outside	7	Staphylococcus pettenkoferi
11	syringe outside	1	Micrococcus luteus
11	syringe outside	3	Staphylococcus chromogenes
11	syringe outside	1	Staphylococcus xylosus
11	syringe outside	4	Staphylococcus pettenkoferi
11	syringe outside	5	Staphylococcus haemolyticus
11	syringe outside	6	Staphylococcus pettenkoferi
11	syringe outside	4	Staphylococcus cohnii
11	syringe outside	4	Staphylococcus pettenkoferi
11	guide wire tip	3	Staphylococcus pettenkoferi
11	guide wire tip	1	Staphylococcus xylosus
11	guide wire tip	2	Staphylococcus chromogenes
11	guide wire tip	3	gram-positive cocci
11	catheter end	2	Staphylococcus haemolyticus
11	catheter end	2	gram-positive cocci
11	catheter end	10	Staphylococcus pettenkoferi
11	catheter end	4	Staphylococcus pettenkoferi
12	working bowl end	2	Staphylococcus haemolyticus
12	working bowl end	3	Staphylococcus chromogenes
12	working bowl end	4	Staphylococcus pettenkoferi
12	working bowl end	1	Kocuria palustris
12	syringe inside	1	gram-positive cocci
12	syringe outside	2	Micrococcus luteus
12	syringe outside	1	Staphylococcus haemolyticus
12	syringe outside	1	gram-positive cocci
12	syringe outside	1	gram-positive cocci
12	catheter tip	1	gram-positive cocci
12	guide wire end	1	Micrococcus luteus
13	working bowl end	2	gram-positive cocci
13	working bowl end	2	Micrococcus luteus
13	working bowl end	2	gram-positive cocci
13	working bowl end	3	Staphylococcus epidermidis
13	working bowl end	1	gram-positive cocci
13	syringe outside	2	Staphylococcus epidermidis
13	syringe outside	1	Micrococcus luteus
13	syringe outside	1	Micrococcus luteus
13	syringe outside	5	Staphylococcus epidermidis
13	syringe outside	1	Micrococcus luteus
13	syringe outside	1	Micrococcus luteus
13	syringe outside	1	Micrococcus luteus
13	guide wire tip	1	Paracoccus yeei
14	working bowl end	1	Staphylococcus capitis

14	syringe outside	2	Micrococcus luteus
14	syringe outside	1	Staphylococcus Capitis
14	syringe outside	1	gram-positive cocci
14	syringe outside	1	Staphylococcus warneri
14	syringe outside	2	Micrococcus luteus
14	syringe outside	2	Moraxella osloensis
14	syringe outside	8	Staphylococcus haemolyticus
14	syringe outside	2	gram-positive cocci
15	working bowl beginning	2	Staphylococcus chromogenes
15	working bowl end	3	Staphylococcus capitis
15	working bowl end	1	Staphylococcus pettenkoferi
15	control bowl end	1	Moraxella osloensis
15	control bowl end	1	Corynebacterium confusum
15	syringe outside	1	Micrococcus luteus
15	syringe outside	2	Micrococcus luteus
15	syringe outside	2	Moraxella osloensis
15	syringe outside	1	Moraxella osloensis
15	syringe outside	1	gram-negative rods
15	syringe outside	5	Corynebacterium spp.
15	syringe outside	2	Micrococcus luteus
15	syringe outside	10	gram-positive rods
15	syringe outside	1	Lysinibacillus spp.
15	syringe outside	14	Staphylococcus pasteuri
15	syringe outside	1	Corynebacterium spp.
15	syringe outside	1	Moraxella osloensis
15	syringe outside	1	Moraxella osloensis
15	syringe outside	4	Micrococcus luteus
15	syringe outside	1	gram-positive cocci
15	guide wire tip	1	Roseomonas mucosa
16	working bowl beginning	1	Staphylococcus capitis
16	working bowl beginning	4	Staphylococcus capitis
16	working bowl beginning	2	Staphylococcus chromogenes
16	working bowl end	1	Staphylococcus capitis
16	working bowl end	4	Staphylococcus chromogenes
16	syringe inside	1	Staphylococcus capitis
16	syringe outside	7	Staphylococcus capitis
16	syringe outside	3	Corynebacterium spp.
16	syringe outside	1	Staphylococcus spp.
16	syringe outside	49	Staphylococcus haemolyticus
16	syringe outside	2	Staphylococcus simulans
16	syringe outside	1	Micrococcus luteus
16	syringe outside	6	Staphylococcus epidermidis
16	syringe outside	12	Staphylococcus epidermidis
16	syringe outside	1	gram-negative rods
16	catheter tip	1	Staphylococcus epidermidis
17	working bowl beginning	1	Staphylococcus chromogenes
17	working bowl end	6	Micrococcus luteus
17	control bowl end	1	Bacillus pumilus
17	syringe inside	3	Bacillus pumilus
17	syringe inside	4	Staphylococcus haemolyticus

17	syringe inside	1	Staphylococcus epidermidis
17	syringe inside	2	Staphylococcus cohnii
17	syringe inside	1	Staphylococcus chromogenes
17	syringe inside	1	Bacillus pumilus
17	syringe inside	1	Staphylococcus capitis
17	syringe inside	1	gram-positive cocci
17	syringe inside	1	Staphylococcus xylosus
17	syringe inside	2	Bacillus pumilus
17	syringe inside	1	Bacillus pumilus
17	syringe inside	1	Moraxella osloensis
17	syringe inside	3	Moraxella osloensis
17	syringe outside	2	Bacillus pumilus
17	syringe outside	1	Staphylococcus epidermidis
17	syringe outside	1	Staphylococcus capitis
17	syringe outside	1	Micrococcus luteus
17	syringe outside	2	Bacillus pumilus
17	syringe outside	4	Staphylococcus capitis
17	syringe outside	1	Staphylococcus epidermidis
17	catheter end	1	Staphylococcus capitis
17	guide wire end	1	Micrococcus luteus
18	working bowl beginning	2	Staphylococcus hominis
18	syringe outside	1	Micrococcus luteus
18	syringe outside	1	gram-negative rods
18	syringe outside	1	gram-negative rods
18	catheter tip	1	Staphylococcus hominis
18	catheter tip	1	Staphylococcus pettenkoferi
18	guide wire tip	1	Micrococcus luteus
